# ﻿*Tachysurustaeniatus* (Günther, 1873), a senior synonym of the congeneric species *T.ondon* (Shaw, 1934) (Teleostei, Bagridae) from eastern China

**DOI:** 10.3897/zookeys.1218.135630

**Published:** 2024-11-20

**Authors:** Wei-Han Shao, Jian-Li Cheng, E. Zhang

**Affiliations:** 1 Institute of Hydrobiology, Chinese Academy of Sciences, Wuhan, 430072, Hubei, China Chinese Academy of Sciences Wuhan China; 2 School of Life Sciences and key laboratory of Jiangxi Province for Biological Invasion and Biosecurity, Jinggangshan University, Ji’an, 343009, Jiangxi, China Jinggangshan University Ji'an China

**Keywords:** Coloration polymorphism, ontogenetic colouration change, original description, pectoral-fin spine, *T.aurantiacus* group, taxonomy

## Abstract

Despite the current recognition of *Tachysurustaeniatus* and *T.ondon* as two separate valid species of China, neither species have been revised based on examination of their types and/or topotypical materials, nor have they genetically analyzed. In this study, examination of the holotype of *T.taeniatus* showed that it has a serrated anterior edge of the pectoral spine, a slightly emarginate caudal fin, and longer maxillary barbels extending beyond the base of the pectoral spine, the characters shared with specimens currently identified as *T.ondon*. Morphological comparisons and molecular analysis showed that specimens from mainland China, which are characterized by the three mentioned morphological features, represent a single species. According to the nomenclatural rule of priority, *T.taeniatus* is a senior subjective synonym of *T.ondon*. Within this concept, *T.taeniatus* is widely distributed in the lower reaches of Yangtze River and coastal rivers in Zhejiang and Fujian Province and closely related to *T.aurantiacus*, which is endemic to Japan. The morphological differences and species-level genetic distance between *T.taeniatus* and *T.aurantiacus* provide additional support for synonymization of *T.taeniatus* and *T.ondon*. The paper also describes ontogenetic color changes and coloration polymorphism in this species. Phylogeny of the *T.aurantiacus* group, to which *T.taeniatus* belongs, is also discussed.

## ﻿Introduction

The bagrid genus *Tachysurus* is a species-rich group comprising more than seventy nominal species, widespread in the Far East to Southeast Asia (Shao and Zhang 2023). Currently, more than 30 of them are considered either invalid or questionable (Fricke et al. 2024). The majority of *Tachysurus* species, as listed by Ferraris (2007) were described more than a century ago. Original descriptions of some species were vague and/or inaccurate; thus, they are either out of modern taxonomic use or misleading when their diagnoses are ambiguous. Despite the recognition of some species as valid in the current taxonomy of *Tachysurus*, they are only known from their original descriptions or by type specimens. One of them is *T.taeniatus*, a species originally described by Günther (1873) based on a single specimen six inches long (about 150 mm) collected from “Shanghai”, without indicating an exact locality.

The original description of *T.taeniatus* mentioned a broad, blackish band along the side of the body and an adipose fin shorter than the anal fin in their basal lengths, both characters distinguishing it from all congeneric species with the rounded caudal fin. Although a recent examination (Watanabe and Kitabayashi 2001) of its holotype showed that the species has small serrations on the anterior margin of the pectoral spine similar to *T.ondon*, this character was not mentioned in the original description of *T.taeniatus*. Chinese researchers (Zheng and Dai 1999) did not have access to the type specimens of many species in their taxonomic revision of this genus. As a result, *T.taeniatus* is recognized as a valid species with a smooth anterior margin of the pectoral spine in the current taxonomy of Chinese *Tachysurus* species (Zheng and Dai 1999). Moreover, no additional specimens identified as *T.taeniatus* have been known from the type locality or adjacent areas since the original description. So, clarification of the taxonomic status of this species has been needed.

*Tachysurusondon* was described by Shaw (1930) based on a single specimen of 77 mm SL captured from the Cao’e-Jiang, a coastal river that ultimately joins the Qiantang-Jiang before flowing into East China Sea, in Shing-Tsong (now Xinchang County in Zhejiang Province). It has so far been identified as a species with a serrated anterior edge of the pectoral spine, widely distributed in montane streams of Southeast China (Zheng and Dai 1999), despite no indication of this character in the original description. Our preliminary observations of Chinese *Tachysurus* species revealed that the anterior edge of the pectoral spine is smooth in all species with emarginate or rounded caudal fins except for *T.ondon* and *T.taeniatus*, as defined herein. Thus, given their similar morphology and proximate type localities, we undertook this study with the goal to clarify the taxonomic status of *T.ondon* and *T.taeniatus* based on morphological examination of the type and/or topotypical specimens integrating morphological and molecular evidence.

## ﻿Materials and methods

### ﻿Species collection and preservation

**Figure 1.**
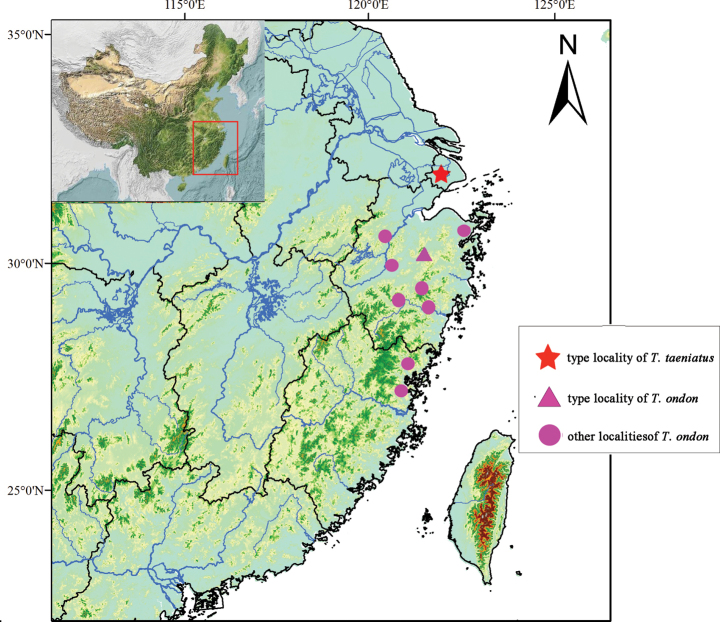
Map showing distributions of *Tachysurustaeniatus* and *T.ondon*.

**Figure 2.**
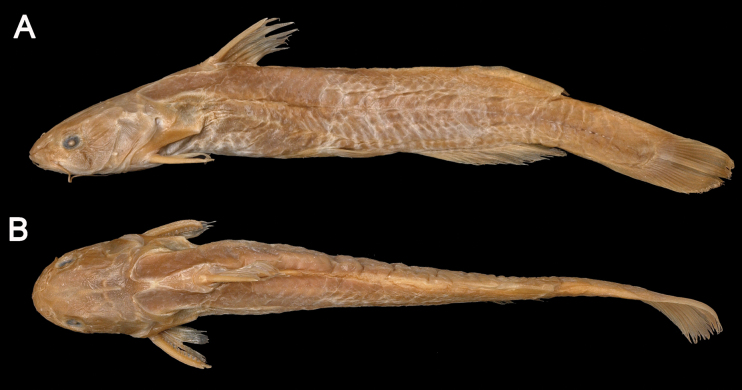
*Tachysurustaeniatus*, holotype, BMNH 1873-7-30-73, 150 mm SL, from Shanghai, China **A** lateral view **B** dorsal view.

Four specimens of *Tachysurustaeniatus* were collected from an affluent of Taihu Lake in Huzhou City, Zhejiang Province. They are thus considered topotypical specimens as their collection site is very close (ca 50 km) to its type locality. Fourteen specimens of *T.ondon* were collected from the Cao’e-Jiang in Xinchang County (type locality), Zhejiang Province. In addition, 64 specimens identified as this species were captured from ten locations (Fig. 1) of coastal rivers in Zhejiang and Fujian Provinces. Captured specimens were stored in 10% formalin liquid after removal of right-side pelvic-fin clips. The extracted fin clips were kept in 95% ethyl alcohol and used for molecular analysis. The voucher specimens are preserved in the ichthyological collection at the Institute of Hydrobiology (**IHB**), Chinese Academy of Sciences, Wuhan. Careful morphological examination was made in this study on the holotype of *T.taeniatus*, currently stored in the Lake Biwa Museum, Japan (Fig. 2A, B).

### ﻿Morphological analysis

Measurements were conducted using digital calipers, with data recorded to the nearest 0.1 mm. Whenever feasible, measurements were taken on the left side of each individual, following the techniques outlined by Cheng et al. (2008). Head length and measurements of other parts of the body are estimated as percentages of the standard length (SL). Subunits of the head are provided as percentages of the head length (HL). The number of rays in the dorsal and anal fins was determined following the method described by Watanabe (1995). Other fin rays were counted under a binocular dissecting microscope using transmitted light. Vertebral count was taken from X-ray photographs, with the five anteriormost vertebrae, namely the Weberian complex, not counted.

Morphometric data underwent principal component analysis (PCA) to reveal variations and assess relative contribution of specific variables to morphometric differences between the species. PCA was run with SPSS 16 (SPSS, Chicago, IL, USA). Before conducting the analysis, all measurements were standardized according to Reist (1985) to avoid effects of allometry.

### ﻿Phylogenetic analysis

Phylogenetic analysis was performed using mtDNA *cyt-b* gene and the sequences have been uploaded to NCBI GenBank (Table 1). Twenty-seven *cyt-b* gene sequences amplified from 17 species of *Tachysurus* were used for molecular phylogenetic analysis. *Tachysurustrilineatus* was selected as the outgroup due to its identification as the basal lineage within the genus *Tachysurus* (Ku et al. 2007). The sequences were manually revised and then aligned using ClustalW in MEGA7 (Kumar et al. 2016). Both Bayesian-inference (BI) and maximum-likelihood (ML) methods were used in the phylogenetic analysis. The optimal nucleotide substitution model was selected by ModelFinder (Kalyaanamoorthy et al. 2017) according to the Akaike Information Criterion. ML analysis was performed using IQ-tree (Nguyen et al. 2015), with the selected TIM3+F+I+G4 model and 1,000 non-parametric bootstrap replicates. Bayesian Inference was performed in MrBayes (Ronquist et al. 2012) under the selected GTR+F+I+G4 model. Two independent runs were carried out with four Monte Carlo Markov chains (three hot chains and one cold chain) for 20 million generations to calculate posterior probability. Trees were sampled every 1000 generations. The initial 25% of sampled trees were discarded as burn-in. Convergence of the runs was assessed by the average standard deviation of split frequencies (<0.01). The genetic distances, based on *cyt-b*, were computed in MEGA 7 using the Kimura-2-parameter (K2P) model (Kimura 1980).

**Table 1.** GenBank accession numbers for molecular phylogenetic analysis.

	Taxon	Locality	Distribution	Accession number
**Ingroup**				
(1)	*Tachysurusaurantiacus*	Japan	Western Kyushu Island	LC533351
(2)	*Tachysurusbrachyrhabdion*	Guizhou, China	Yuan-Jiang of middle Yangtze River	PP266650
(3)	*Tachysurusbrevicorpus*	South Korea		NC_015625
(4)	*Tachysuruseupogon*	Hubei, China	Middle Yangtze River	PP266669
(5)	*Tachysurusgracilis*	Guangxi, China	Xiang-Jiang of middle Yangtze River	PP266654
(6)	*Tachysurusintermedius*	Hainan, China	Nandu-Jiang	PP266676
(7)	*Tachysuruskoreanus*	South Korea		NC028434
(8)	*Tachysuruskyphus*	Guangxi, China	Fangcheng- Jiang	PP266671
(9)	*Tachysuruslongispinalis*	Vietnam	Red River	PP266672
(10)	*Tachysurusnudiceps*	Japan	Central Honshu, Shikoku and eastern Kyushu Islands	LC664019
(11)	“*Tachysurusondon*” NINGB37529	Zhejiang, China	Qiantang-Jiang	PQ497556
	“*Tachysurusondon*” NINGB37531	Zhejiang, China	Qiantang-Jiang	PQ497557
	“*Tachysurusondon*” NINGB37534	Zhejiang, China	Qiantang-Jiang	PQ497558
	“*Tachysurusondon*” LIANJ30623	Fujian, China	Ao-Jiang	PQ497555
	“*Tachysurusondon*” NINGD13612	Fujian, China	Jiao-Xi	PQ497559
	“*Tachysurusondon*” NINGD13614	Fujian, China	Jiao-Xi	PQ497560
	“*Tachysurusondon*” QINGT35898	Zhejiang, China	Ou-Jiang	PQ497561
	“*Tachysurusondon*” XINC66761	Zhejiang, China	Cao’e-Jiang	PQ497562
	“*Tachysurusondon*” XINC66762	Zhejiang, China	Cao’e-Jiang	PQ497563
(12)	*Tachysuruspratti*	Fujian, China	Upper Yangtze River	PP266656
(13)	*Tachysurussinensis*	Sichuan, China	Middle Yangtze River	PP266674
(14)	*Tachysurustaeniatus* 1	Zhejiang, China	Taihu Lake	PQ497552
	*Tachysurustaeniatus* 2	Zhejiang, China	Taihu Lake	PQ497553
	*Tachysurustaeniatus* 3	Zhejiang, China	Taihu Lake	PQ497554
(14)	*Tachysurustokiensis*	Japan	Eastern Honshu Island	AB054127
(15)	*Tachysurustruncatus*	Sichuan, China	Upper Yangtze River	PP266658
(16)	*Tachysurusvirgatus*	Hainan, China	Jiajihe River	PP266673
**Outgroup**				
(17)	*Tachysurustrilineatus*	Guangdong, China	Dong-Jiang of Pearl River	PP266679

## ﻿Results

### ﻿Examination on the holotype of *T.taeniatus*

**Figure 3.**
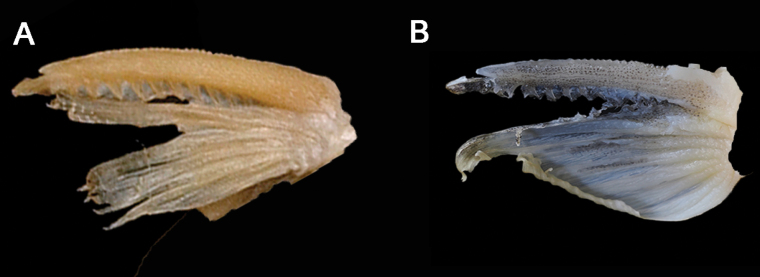
Serrations on the anterior edge of pectoral spine **A** holotype of *Tachysurustaeniatus* (BMNH 1873-7-30-73, 150 mm SL, from Shanghai, China) **B** topotype of *T.ondon* (IHB081570, 81.2 mm SL, from Xinchang County, Zhejiang Province).

The holotype of *Tachysurustaeniatus*, currently stored in the British Museum of Natural History (BMNH), had not been examined by any Chinese investigators before this study. Our observation of this holotype (BMNH 1873-7-30-72; Fig. 2) coincides with its original description in the following characters: (1) maxillary barbels extending beyond the insertion of the pectoral fin, (2) nasal barbels extending beyond the posterior edge of the eye, (3) an adipose-fin shorter than the anal-fin in basal length, (4) a dorsal spine shorter than the body and head depth. However, the holotype has a slightly emarginate caudal fin with the upper lobe slightly longer than the lower lobe (Fig. 2B), rather than a rounded one as stated in its original description. Additionally, it also possesses small serrations on the anterior edge of the pectoral spine covered with skin, a character not mentioned in the original description but shared with the topotypical specimens of *T.ondon* (Fig. 3).

### ﻿Note on type locality of *Tachysurustaeniatus*

The original description (Günther 1873: 240) is based on a specimen from a collection of freshwater fishes from China sent to the British Museum by Robert Swinhoe, H.M. Consul at Shanghai, who collected the fish “at that place” Günther 1873: 240); no specified localities are given. Though, some materials could be collected by Swinhoe during his trip upstream the Yangtze River in 1869 (Heok Hee Ng personal communication), we suppose that *Tachysurustaeniatus* was found in Shanghai or nearby area because of the following reasons. First, the species hasn’t been collected from Shanghai in recent years due to urbanization; however, it still occurs in areas close to Shanghai, such as Huzhou City in Zhejiang Province. Second, and more important, *T.taeniatus* shows coloration polymorphism, and the coloration pattern described in the original publication—a continuous black stripe along the mid-body—is only found in Shanghai and some locations in adjacent Zhejiang Province (belonging to coastal rivers of southeast China); this particular coloration is absent in *T.taeniatus* from other parts of the Yangtze River.

### ﻿Body coloration in topotypical specimens *Tachysurustaeniatus* and *T.ondon*

**Figure 4.**
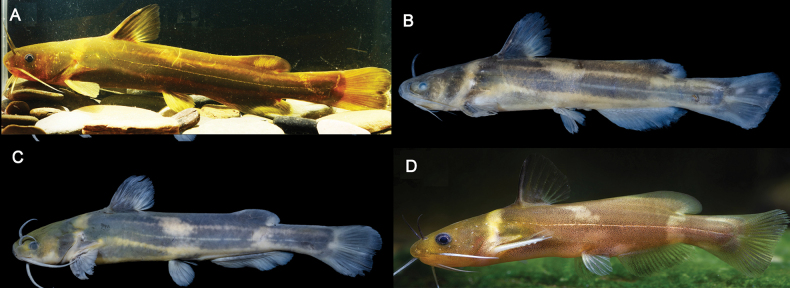
**A** color in life of adult of topotype of *Tachysurustaeniatus***B** lateral view of *T.ondon*, topotype, IHB081570, 81.2 mm SL, Xinchang county, Zhejiang Province **C** lateral view of *T.taeniatus*, topotype, IHB202406066714, 74.4 mm SL, Huzhou city, Zhejiang Province **D** lateral view of a live specimen of *T.ondon* collected from Ningde City, Fujian Province.

Brief accounts on the coloration pattern of the two species were provided in their original descriptions based only on a single specimen for each species, making it unfeasible to understand the intraspecific variations of coloration. In the *T.taeniatus* and *T.ondon* specimens examined in this study, ontogenetic changes in coloration were observed: the lateral blackish band or blotches found in small individuals becomes blurred in adult individuals exceeding 180 mm SL. Moreover, three coloration morphs exist in the juveniles and subadults of the specimens of these two species. One morph includes individuals which possess a yellowish body with three longitudinal blackish bands along the lateral body, of which the median band is continuous but the other two are interrupted to form three rectangular blotches (Fig. 4A). This coloration morph, as stated in the original description of *T.taeniatus* which reads “a broad blackish band along the side of the body”, is only found in some topotypical specimens (Günther 1873). Despite the shared presence of three blackish bands, another coloration morph develops an uninterrupted median band. This is also present in the topotypical specimens of both *T.taeniatus* and *T.ondon* (Fig. 4B, C). Furthermore, one more morph was detected in the specimens identified as *T.ondon* from Ningde City, Fujian Province with three broad vertical brown blotches on the yellowish background of the body (Fig. 4D).

### ﻿Morphometric and meristic comparisons between *Tachysurustaeniatus* and *T.ondon*

The specimens designated for comparative analysis were categorized into three groups: 1. topotypic *T.taeniatus*, 2. topotypic *T.ondon*, 3. other specimens primarily identified as *T.ondon*. The measurements of the examined specimens are summarized in Table 2. No discrete differences between these groups were found by comparing the morphometric data. Meristic counts for the type specimens of *T.taeniatus* and *T.ondon*, and other specimens examined of *T.ondon* are given in Table 3. Three meristic characters have variable counts, namely anal and pelvic fins, and vertebrae. Counts of all three meristic characters are not significantly different with overlapping ranges among the three groups.

**Table 2.** Morphomertric data for *Tachysurustaeniatus* and *T.ondon*.

	T.taeniatus		T.ondon			
	Topotypes (*n* = 4)		Topotypes (*n* = 14)		Other specimens (*n* = 64)	
	Range	Mean ± SD	Range	Mean ± SD	Range	Mean ± SD
Standard length	66.9–149.9	106.4	43.1–118.3	80.5	55.79–142.2	87.9
In SL (%)						
Body depth at anus	13.6–17.3	15.4	14.7–18.0	16.1	12.5–17.8	15.3
Predorsal length	32.0–38.3	34.5	31.0–37.3	34.4	30.3–39.6	34.3
Pre-anal length	57.0–62.3	59.5	56.5–67.5	62.1	57.4–65.9	62.3
Prepelvic length	47.0–52.6	49.4	45.1–56.6	50.7	46.4–55.1	50.9
Length of dorsal-fin spine	12.6–18.2	15.7	10.4–18.4	14.5	13.3–18.6	16.8
Length of dorsal-fin base	12.1–14.5	13.4	10.1–15.1	11.9	12.7–15.0	13.8
Length of pectoral-fin spine	11.3–18.5	15.1	10.8–19.3	15.1	14.2–18.1	16.2
Length of anal-fin base	21.4–24.3	22.6	18.2–25.2	22.9	20.2–25.1	22.9
Adipose to caudal distance	16.1–17.3	16.9	14.1–18.4	15.9	14.6–19.1	16.9
Length of caudal peduncle	16.4–18.0	16.9	14.5–18.5	16.2	14.7–20.2	17.1
Depth of caudal peduncle	8.5–9.4	9.0	8.0–10.2	8.9	7.4–9.9	8.6
Head length at latera	22.9–25.2	24.0	24.5–30.7	26.5	22.3–28.2	24.8
In HL (%)						
Head depth	55.5–64.0	59.9	50.0–60.3	55.2	44.9–56.6	52.4
Head width	71.2–82.6	77.2	64.1–77.8	71.1	64.0–76.1	72.5
Snout length	23.1–31.0	27.5	22.9–33.6	26.9	25.8–32.7	27.9
Interorbital width	40.1–51.3	44.9	35.0–44.7	39.6	39.1–48.6	41.0
Eye diameter	14.8–21.7	18.3	14.2–25.8	19.9	14.6–22.5	17.7
Mouth width	52.5–60.4	56.5	47.0–57.3	50.7	46.5–58.7	50.1
Length of nasal barbel	43.6–57.2	51.3	41.0–54.8	47.0	41.5–57.3	49.5
Length of maxillary barbel	90.1–105.2	97.5	77.7–98.5	87.4	81.5–121.2	96.1
Length of inner mandibular barbel	44.0–54.4	48.2	41.7–55.9	48.1	35.1–55.3	44.1
Length of outer mandibular barbel	66.4–75.2	69.0	61.0–78.9	70.9	56.4–79.0	67.5

**Table 3.** Meristic counts (mean ± SD) for *Tachysurustaeniatus* and *T.ondon*.

	*T.taeniatus*	*T.ondon*	
	Topotypes	Tototypes	Other specimens
Soft rays			
Dorsal	7(7 ± 0.0)	7(7 ± 0.0)	7(7 ± 0.0)
Anal	17–19(18.5 ± 0.4)	18–20(19.2 ± 0.5)	16–20(18.1 ± 1.3)
Pectoral	7(7 ± 0.0)	7(7 ± 0.0)	7(7 ± 0.0)
Pelvic	6(6 ± 0.0)	6(6 ± 0.0)	6–7(6.2 ± 0.1)
Vertebrae	39–42(40.1 ± 1.8)	40–43(41.2 ± 1.1)	39–44(41.8 ± 2.1)

**Figure 5.**
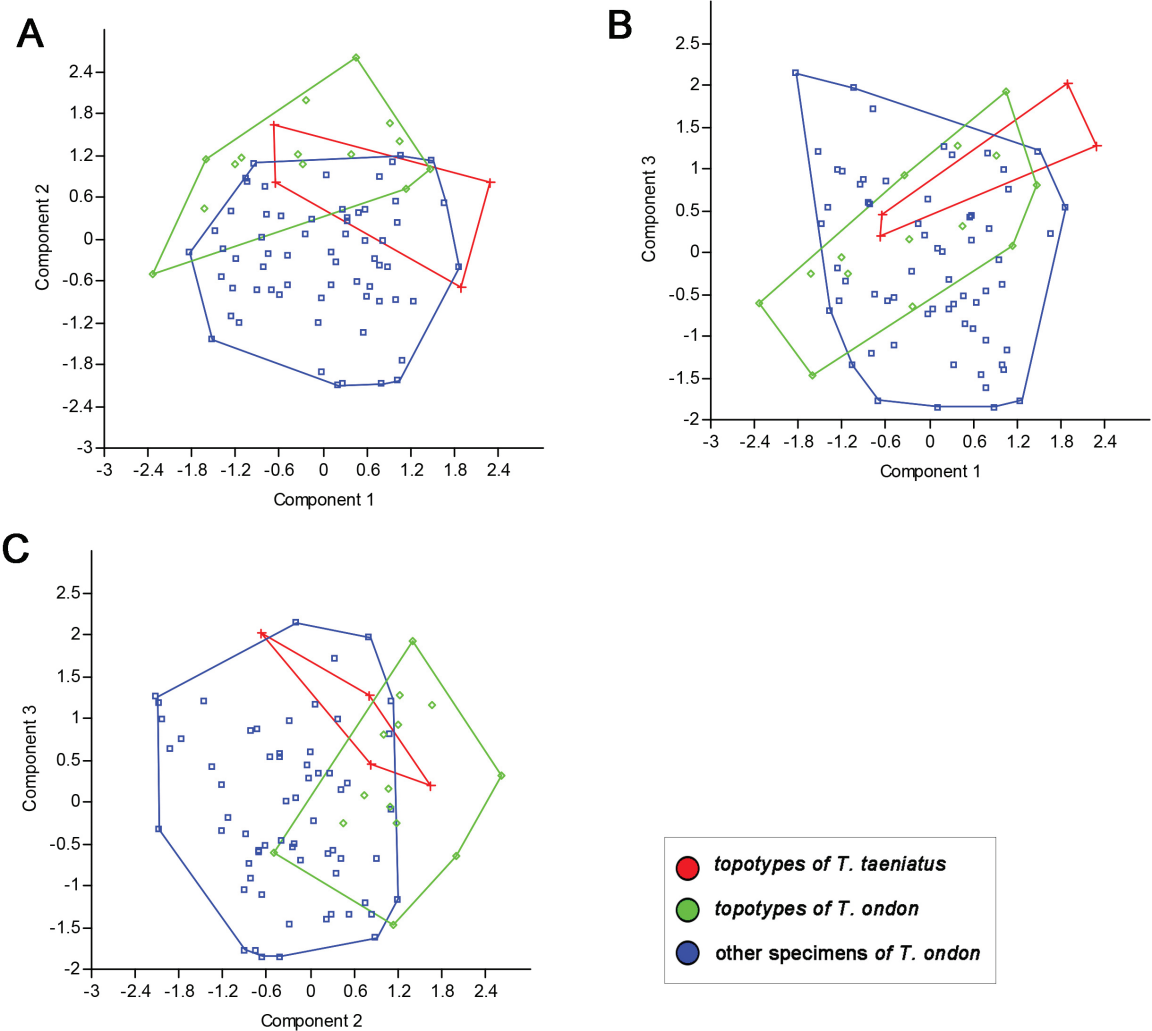
Scatter plots **A** PC1 against PC2 **B** PC1 against PC3 **C** PC2 against PC3 extracted from morphometric data for examined materials of *T.taeniatus* and *T.ondon*.

In a principal component analysis (PCA) performed on twenty-two morphometric characters, the combinations of PC1 against PC2 and PC3, along with PC2 against PC3, failed to separate the examined specimens of *T.ondon* and the topotypes of *T.taeniatus* (Fig. 5).

### ﻿Molecular comparisons

**Figure 6.**
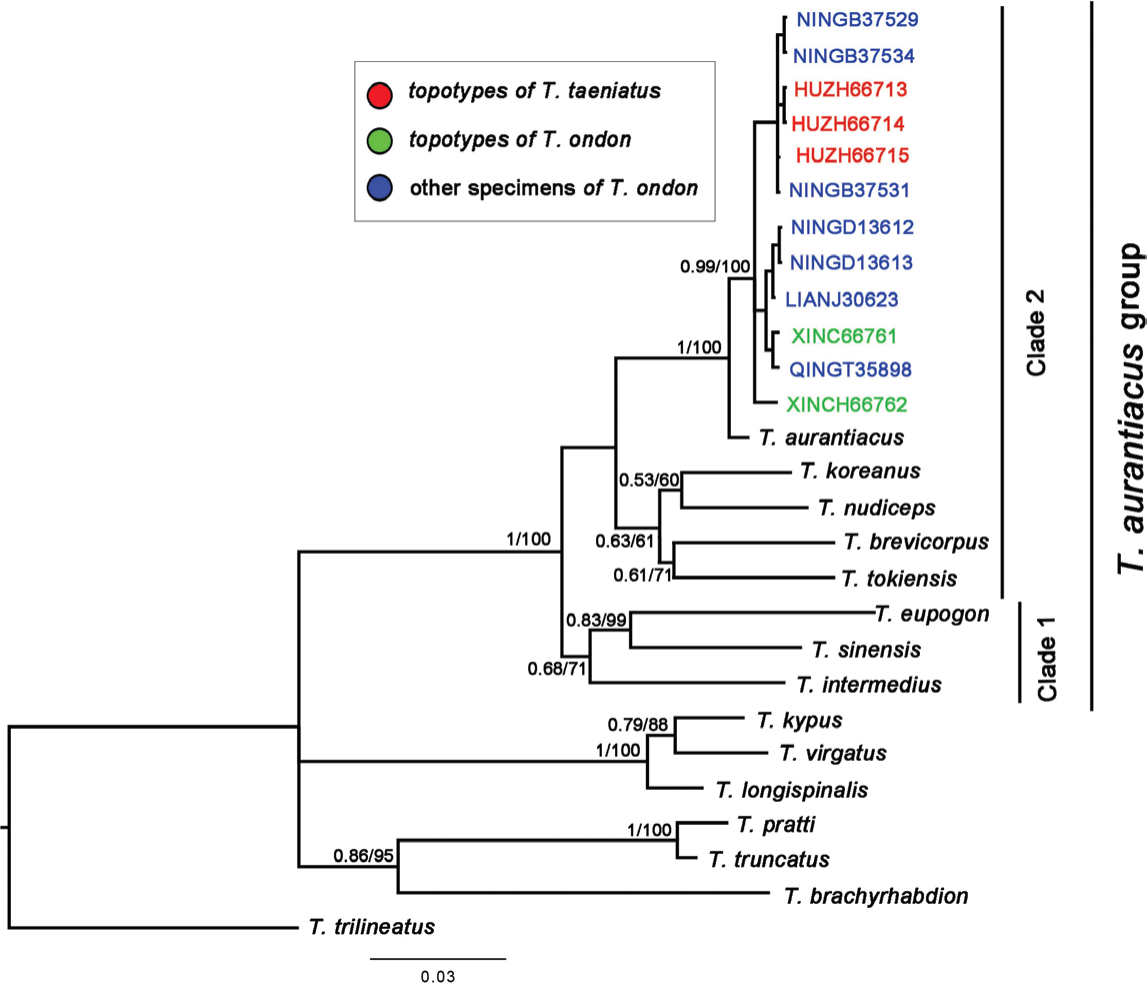
Phylogenetic tree of *Tachysurus* species inferred from *cyt-b* using Bayesian-inference and maximum-likelihood methods. Bayesian posterior probabilities (>0.6) and maximum-likelihood bootstrap values (>60%) are shown, respectively. All members of the *T.aurantiacus* group (as defined by Shao and Zhang 2023), with the exception of *T.brevianalis*, were included in the phylogenetic tree.

A total of 1066 bps were included in the aligned dataset of the *cyt-b* gene, with 642 conservative sites, 424 variable sites, 242 parsim-informative sites and 94 singleton sites. The topologies of the phylogenetic trees were found to be similar between ML and BI methods (Fig. 6). The three topotypical specimens of *T.taeniatus* were found to be together with the specimens examined of *T.ondon* in a monophyletic group that was recovered with 100% posterior probabilities (pp) and 0.99 bs in ML and BI trees, respectively. This monophyly showed a phylogenetic affinity to the Japan endemic *T.aurantiacus*, within a clade containing *T.koreanus*, *T.nudiceps*, *T.brevicorpus*, *T.tokiensis*, *T.eupogon*, *T.sinensis* and *T.intermedius*, all of which were designated as belonging to the *T.aurantiacus* group by Shao and Zhang (2023).

The two closely related species of *T.taeniatus* and *T.aurantiacus* have a genetic distance of 1.5% and the distances between *T.taeniatus* and other members of the *T.aurantiacus* group are in the range of 6.4–10.0% (Table 4).

**Table 4.** K2P distances (%) for species within the *Tachysurusaurantiacus* group, based on the *cyt*-b gene.

	1	2	3	4	5	6	7	8
*T.taeniatus*								
*T.aurantiacus*	1.5							
*T.koreanus*	6.5	5.9						
*T.nudiceps*	6.4	5.8	4.8					
*T.brevicorpus*	7.7	7.2	6.1	6.2				
*T.tokiensis*	6.9	6.9	5.8	6.0	6.9			
*T.eupogon*	10.0	9.6	9.3	9.4	8.9	11.0		
*T.sinensis*	8.1	7.8	8.5	8.6	8.8	8.2	8.2	
*T.intermedius*	8.0	7.4	8.2	7.3	9.8	7.8	9.1	8.1

## ﻿Discussion

### ﻿Identity of *Tachysurustaeniatus*

Main distinguishing characters of *Tachysurustaeniatus*, as herein diagnosed, include: (1) a serrated anterior edge of the pectoral spine; (2) a slightly emarginate caudal fin with the upper lobe slightly longer than the lower lobe; (3) longer maxillary barbels extending beyond the base of the pectoral spine; and (4) a shorter dorsal-fin spine than body depth. The first character was not mentioned in the original description. Our examination of the holotype confirmed observation by Watanabe and Kitabayashi (2001) that *T.taeniatus* has a serrated anterior edge of the pectoral spine (Fig. 3A). The statement of the second character (caudal-fin shape) in the original description of this species is inaccurate. According to our examination of the holotype, *T.taeniatus* has a slightly emarginate caudal fin with an upper lobe slightly longer than the lower lobe, in contrast with its original description that reads: “Caudal rounded”. As far as is known, rounded caudal fins are only present in *T.tenuis*, *T.trilineatus*, *T.analis*, and *T.lani* (Cheng et al. 2021; Shao et al. 2021). Clearly, misled by the vagueness and inaccuracy of its original description and no accessibility to type materials, subsequent Chinese researchers (Zheng and Dai 1999) had a misconception that *T.taeniatus*, thus erroneously identify it as a species with a smooth anterior margin of the pectoral spine and a rounded caudal fin. This can reasonably explain why no additional specimens of *T.taeniatus* have been found from its type locality and nearby river systems since the original description.

### ﻿Synonymization of *Tachysurustaeniatus* and *T.ondon*

Both *T.taeniatus* and *T.ondon* are currently assigned to the *T.aurantiacus* group which, thus, included eleven species (Shao and Zhang 2023). Four species, namely *T.nudiceps*, *T.sinensis*, *T.eupogon*, and *T.intermedius*, develop deeply forked caudal fins. A slightly emarginate caudal fin is found in the remaining seven species, viz., *T.aurantiacus*, *T.koreanus*, *T.tokiensis*, *T.brevianalis*, *T.brevicorpus*, *T.taeniatus*, and *T.ondon*. The last two species are known only from the mainland China, with the other species occurring in Korea Peninsula, Japan Archipelago, and Taiwan Island. However, our molecular phylogenetic analysis based on the *cyt-b* gene showed that all samples identified as these two species from Zhejiang and Fujian provinces of China, including those from the type locality of *T.ondon* and an affluent of Lake Taihu in proximity to the type locality of *T.taeniatus*, clustered together into a lineage sister to *T.aurantiacus*, a species endemic to western Kyushu of Japan (Watanabe and Maeda 1995).

Despite a 1.5% genetic distance between *T.taeniatus* (including *T.ondon*) and *T.aurantiacus*, they are clearly distinguishable by the snout length, 33–41% HL vs 23–33% (data for *T.aurantiacus* from Watanabe and Maeda (1995) and serration on the anterior edge of the pectoral spine which is better developed in *T.aurantiacus* incomparison with *T.taeniatus*. Although, the genetic distances between the two taxa are comparatively low, it lies within the range used for delineating currently described species of *Tachysurus*, for example, the genetic distance between *T.pratti* and *T.truncatus* is 1.4% and between *T.longispinalis* and *T.kyphus* is 1.6% (Ku et al. 2007). The relatively low genetic distance between closely related species within *Tachysurus* may be attributed to the significantly low substitution rate of the mitochondrial genes in the East Asian bagrids and the recent estimated divergence time as supposed by Peng et al. (2002), Campbell and Piller (2017) and Shao et al. (2021). Besides, the two species have discontinuous distribution patterns. So, we treat them as two distinct species.

The genetic divergence among samples collected from coastal rivers of Zhejiang and Fujian provinces was 0.3–0.4%. No significant morphological variations were found among different geographic populations. All these findings suggest that the samples from mainland China, with a serrated anterior edge of the pectoral spine and a slightly emarginate caudal fin, represent a single species. According to International Code for Zoological Nomenclature, Art. 23.3 (International Commission on Zoological Nomenclature 1999), *T.taeniatus* should be considered a senior subjective synonym of *T.ondon*.

### ﻿Coloration variation in *Tachysurustaeniatus*

In Chinese literature, a continuous black longitudinal stripe on the lateral body is viewed as the main diagnostic character for *T.taeniatus* (Zheng and Dai 1999). This body coloration pattern was utilized in this study to identify four specimens as *T.taeniatus* collected from an affluent of Lake Taihu, very close to the type locality. Ontogenetic changes in body coloration, though, were detected in *T.taeniatus*; there are conspicuous variations found between juveniles/sub-adults and adults. The blackish stripes or blotches on the flank become less distinct or disappear entirely in individuals of more than 180 mm SL, thus giving a uniformly brown body coloration. A similar ontogenetic change in body coloration has also been documented for closely related species, including *T.aurantiacus*, *T.tokiensis*, and *T.koreanus* (Lee and Kim 1990; Watanabe and Maeda 1995), all of them being members of the *T.aurantiacus* group. Recent studies of catfishes have inferred that ontogenetic coloration transformations are closely related to ontogenetic changes in daily activity period (Zanata and Prmitivo 2013; Vilardo et al. 2020; Costa et al. 2023). Juveniles and subadults exhibit heightened activity during the day, whereas adults display a preference for nocturnal behavior, necessitating a darker coloration phenotype. This explanation aligns with the field evidence presented in this study. Coloration polymorphism was also observed in the juveniles and sub-adults of *T.taeniatus* with three coloration morphs detected (Fig. 2). This implies that the body coloration of this species can vary not only due to ontogenetic changes but also in response to local environmental conditions and geographical distributions.

It is apparent that the existence of ontogenetic coloration changes and coloration polymorphism in *T.taeniatus* and closely related species argue against solely relying on body coloration for distinguishing species of *Tachysurus*. However, it is also incorrect to totally dismiss the taxonomic value of body coloration in *Tachysurus*. For example, *T.trilineatus* has three longitudinal brownish narrow bands running along its flank with the median band featuring a row of yellow spots along the lateral line, a unique body coloration separating it from all congeneric species (Shao and Zhang 2023). This unique body coloration has led to the designation of this species as a monotypic group, a classification supported by a molecular phylogenetic analysis (Ku et al. 2007). Body coloration should not be discarded a priori as evidence of species boundaries within *Tachysurus*. It is essential to reinforce species delineation hypotheses using additional morphological evidence such as morphometric characters or molecular data rather than relying solely on coloration.

### ﻿Phylogenetic structure of *Tachysurusaurantiacus* group

The smoothness of the anterior edge of the pectoral-fin spine has been considered a significant diagnostic character in *Tachysurus* (Ku et al. 2007; Shao and Zhang 2023). The *T.aurantiacus* group, containing nine species (Fig. 6), can be distinguished from the congeners by the presence of a rough pectoral-fin spine (Shao and Zhang 2023). With the exception of *T.brevianalis*, which is endemic to Taiwan Island, all members of this group were included in the phylogenetic analysis in the present study that revealed its monophyly. The group is further subdivided into two clades (Fig. 6) different in some morphological features and ecological niches. Clade 1 includes *T.sinensis*, *T.intermedius*, and *T.eupogon*, distributed in mainland China and northern Vietnam. This clade is characterized by a forked caudal fin and a preference to inhabit the main stream of rivers. The remaining species of *T.aurantiacus* group form clade 2 (*T.taeniatus*, *T.aurantiacus*, *T.koreanus*, *T.nudiceps*, *T.brevicorpus*, *T.tokiensis*). Except for *T.nudiceps*, the members of this clade have round-tailed caudal fins and are adapted to fast-flowing montane streams (Krishnadas et al. 2018). All members of this clade, with the exception of *T.taeniatus*, are distributed in Japan or the Korean Peninsula. Since *T.taeniatus* appears to be the youngest lineage in clade 2, it is likely that the ancestor of clade 2 likely originated in Japan and/or the Korean Peninsula, subsequently dispersed to mainland China during interglacial periods (Zahiri et al. 2019) followed by subsequent isolation resulted in separation of *T.taeniatus* at the species level.

## ﻿Additional information

### Author ORCIDs

E. Zhang https://orcid.org/0000-0002-6971-7160

### Data availability

All of the data that support the findings of this study are available in the main text.
